# Commentary: Immunomodulatory role of gut microbial metabolites: mechanistic insights and therapeutic frontiers

**DOI:** 10.3389/fmicb.2026.1750075

**Published:** 2026-03-30

**Authors:** Zhihui Zeng, Yun Wang, Danqin Fang, Wentao Guo

**Affiliations:** 1 Shenzhen TCM Anorectal Hospital (Futian), Shenzhen, China; 2 Shenzhen Traditional Chinese Medicine Hospital, Shenzhen, China

**Keywords:** gut microbial metabolites, host genetic variability, immunomodulation, mechanistic insights, therapeutic frontiers

## Introduction

Zeng et al. provide a comprehensive synthesis of the burgeoning field of gut microbial metabolites and their immunomodulatory roles, offering a timely exploration of mechanistic pathways and therapeutic potentials. This review is characterized by its ambitious scope in bridging basic science with clinical applications, but its breadth warrants a critical analysis of depth and translational feasibility. The authors highlight how metabolites like short-chain fatty acids (SCFAs) and tryptophan derivatives serve as critical mediators in immune regulation, yet the breadth of this synthesis necessitates a closer examination of its depth and translational feasibility (Rooks and Garrett, 2016). This commentary builds upon the article's strengths while addressing key academic gaps. For instance, while the review adeptly maps canonical signaling pathways, it pays insufficient attention to how host attention to how host genetic polymorphisms (e.g., in AhR or GPR43 receptors) can drastically alter metabolite efficacy across individuals. Furthermore, the discussion on therapeutic frontiers, though forward-thinking, would benefit from a more critical appraisal of hurdles such as the ecological stability of engineered probiotics in heterogeneous gut environments. This commentary will delve into these specific aspects to refine the review's translational framework. By promoting concise discussion, we can refine the trajectory of future research in this dynamic area, ultimately enhancing the precision of microbiome-based interventions.

## Critical assessment of mechanistic insights

Zeng et al. provide a thorough overview of biosynthetic pathways and immunoregulatory mechanisms. However, the discussion would benefit from incorporating context-dependent factors, such as how host genetic polymorphisms (e.g., AhR variants) alter metabolite efficacy, as highlighted in recent studies (Kim, 2021). For instance, the dose-dependent effects of metabolites are highlighted, but the review falls short of exploring how host genetic factors—such as polymorphisms in receptors like AhR or enzymatic pathways—might alter metabolite efficacy or lead to divergent immune outcomes in different populations. This omission is important, as recent studies emphasize that personalized genetic backgrounds can drastically influence metabolite-receptor interactions, potentially explaining why some therapeutic attempts yield inconsistent results in clinical trials (Kim, 2021). For instance, polymorphisms in the AhR gene (e.g., rs2066853) have been shown to modulate the efficacy of tryptophan metabolites in inducing IL-22 production, which could account for variable immune responses in populations with different genetic backgrounds. This genetic variation may underpin the inconsistent clinical outcomes observed in trials of AhR agonists for inflammatory bowel disease, a nuance that is crucial for developing stratified therapies. Similarly, beyond AhR, genetic variations in receptors for other key metabolites warrant consideration. For instance, polymorphisms in the GPR43 (FFAR2) gene, the primary receptor for short-chain fatty acids like butyrate, have been associated with altered immune cell responses and susceptibility to inflammatory conditions. Specific SNPs may affect ligand binding or receptor expression, leading to interindividual differences in the efficacy of SCFA-mediated immunoregulation. This underscores that host genetic variability is not limited to a single pathway but constitutes a broad-spectrum modulator of host-microbiome crosstalk. Integrating such data would elucidate how metabolite signaling is not universal but context-dependent, thereby refining the review's mechanistic framework. Moreover, the article's focus on canonical pathways overlooks emerging mechanisms, such as the role of metabolite-induced epigenetic modifications beyond HDAC inhibition, which could enrich the narrative. Integrating such nuances would mitigate the risk of oversimplification and provide a more holistic view of the metabolite-immune axis.

## Evaluation of therapeutic frontiers and methodological gaps

In discussing therapeutic applications, the authors describe innovative strategies like engineered probiotics and nanomedicine, pointing toward a future of spatiotemporal precision in targeting the gut-immune axis (Agus et al., 2021). The emphasis on AI-driven modeling and multiomics integration is particularly forward-thinking, aligning with current trends in predictive biology. Nonetheless, the review could benefit from a more critical appraisal of formidable translational hurdles. For example, the stability and colonization of engineered probiotics can be compromised by factors such as phage predation, nutrient competition from the resident microbiota, and host immune responses against bacterial vectors—aspects that warrant more critical scrutiny than provided. Additionally, the article would benefit from a sharper focus on the challenges of achieving personalized therapies; although the concept of “dose-time windows” is introduced, there is limited discussion on how to operationalize this in clinical settings, where interindividual microbiome variability is the norm rather than the exception. A deeper engagement with recent work on microbiome resilience (Hezaveh et al., 2022) could illuminate pathways to overcome these barriers and operationalize personalized therapy. For instance, leveraging patient-specific microbiome profiling combined with metabolomic data could inform the design of “dynamic dosing” regimens. A concrete example can be found in recent pilot trials for inflammatory bowel disease, where metagenomic predictors were used to personalize SCFA supplementation schedules, thereby operationalizing the “dose-time window” concept in a clinically actionable manner. Specifically, leveraging patient-specific microbiome profiling combined with metabolomic data could inform the design of “dynamic dosing” regimens that adapt to individual gut environments, as seen in recent trials using metagenomic predictors to optimize SCFA supplementation in inflammatory bowel disease. This approach would address the “dose-time window” concept more operationally, highlighting how computational tools can translate mechanistic insights into tailored therapies.

**Figure 1:**
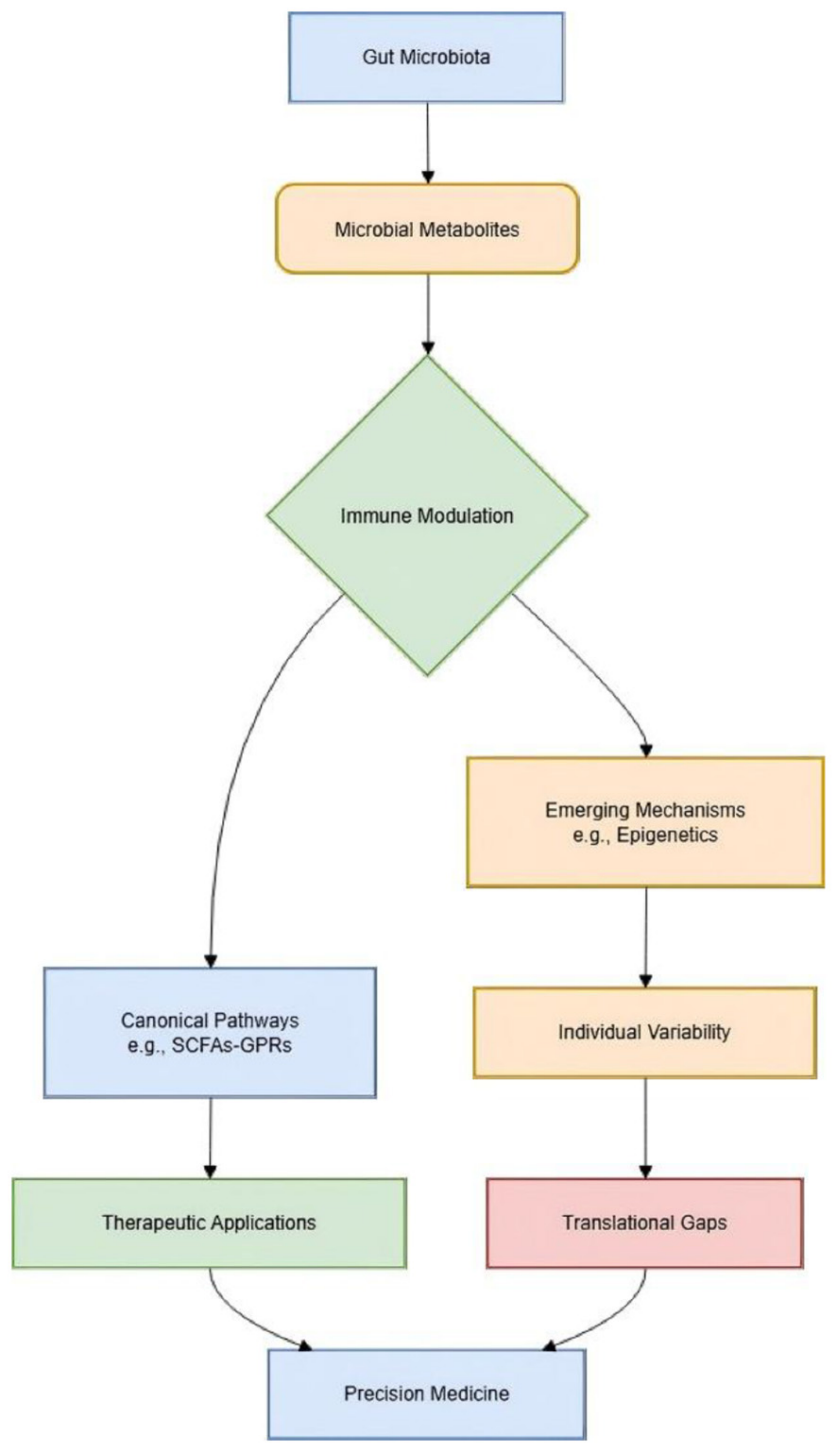
Integrative challenges and future directions in targeting the gut metabolite-immune axis.

Figure 1 delineates two primary pathways:

1. The Mechanistic Pathway (Left): Depicts the canonical sequence from dietary input to immune modulation, wherein dietary components are metabolized by a diverse gut microbiota into key immunoregulatory metabolites (e.g., SCFAs, AhR ligands). These metabolites engage host receptors (e.g., GPCRs, AhR) on epithelial and immune cells, ultimately shaping cytokine profiles and maintaining immune homeostasis.
2. The Translational Challenge Pathway (Right): Highlights areas where the reviewed synthesis could be enhanced, as discussed in your commentary. This includes the influence of host genetic variability (e.g., AhR polymorphisms) on metabolite-receptor efficacy, the ecological resistance of the gut environment to engineered probiotics, and the individualized dosing challenge posed by inter-personal microbiome diversity. These factors converge to create a “translational gap” between mechanistic insight and clinical application.

## Discussion

In summary, Zeng et al. summarize the current knowledge on gut microbial metabolites and immunity. However, the broad scope may limit depth in areas such as host genetic variability, underscoring the need for future longitudinal studies. Moving forward, research must prioritize longitudinal studies in genetically and culturally diverse cohorts, designed to correlate host genotypes (e.g., AhR, GPR43 variants), individual microbiome and metabolome profiles (which are heavily diet-influenced), and clinical outcomes. This tripartite perspective—host genetics, microbiome ecology, and dietary habit—is essential to bridge the translational gap. Computational models integrating these multi-omics layers can predict individual-specific “metabolite-receptor-diet interactomes,” moving the field from one-size-fits-all insights to actionable, personalized therapies. Coupled with advanced computational models, this approach can predict individual-specific “metabolite-receptor interactomes,” moving the field from one-size-fits-all mechanistic insights to actionable, personalized therapies. Addressing these aspects is crucial to translate the promise of microbiome modulation into clinical practice. This commentary underscores the need for a balanced approach that celebrates achievements while conscientiously navigating the intricacies of host-microbe symbiosis.
